# A transcriptomic study of myogenic differentiation under the overexpression of PPARγ by RNA-Seq

**DOI:** 10.1038/s41598-017-14275-2

**Published:** 2017-11-10

**Authors:** Kan He, Guoying Wu, Wen-Xing Li, Daogang Guan, Wenwen Lv, Mengting Gong, Shoudong Ye, Aiping Lu

**Affiliations:** 1 Department of Biostatistics, School of Life Sciences, Anhui University, Hefei, 230601 Anhui China; 2 School of Chinese Medicine, Hong Kong Baptist University, 7 Baptist University Road, Kowloon Tong, Hong Kong China; 3 Center for Stem Cell and Translational Medicine, School of Life Sciences, Anhui University, Hefei, 230601 Anhui China; 4 State Key Laboratory of Genetic Resources and Evolution, Kunming Institute of Zoology, Chinese Academy of Sciences, Kunming, 650223 Yunnan China; 5 Kunming College of Life Science, University of Chinese Academy of Sciences, Kunming, 650204 Yunnan China; 6 Hongqiao International Institute of Medicine, Shanghai Tongren Hospital/Faculty of Public Health, School of Medicine, Shanghai Jiao Tong University, Shanghai, 200025 China

## Abstract

To study the cellular and molecular function of peroxisome proliferator-activated receptor γ (PPARγ) in skeletal muscle differentiation, we have generated inducible gain-of-function to overexpress PPARγ in C2C12 myoblasts. In order to identify PPARγ targets, RNA sequencing (RNA-seq) was used to evaluate and quantify the transcriptomes and expression patterns during myogenic differentiation under the overexpression of PPARγ. The formation of myotubes and the expression of muscle-specific myogenic genes such as *MyoD* and *MyoG* may be inhibited by PPARγ overexpression. Multiple genes and pathways were significantly involved in this process, including 11 genes such as *Fndc9* and *Slc14a1* with fundamental change of regulation modes, 9 genes of which were validated by the data of qRT-PCR. Our studies demonstrate that PPARγ would play critical roles on myoblasts differentiation, mediating crosstalk among several pathways and transcription factors. Our data is available in the Gene Expression Omnibus (GEO) database with the accession number as GSE99399.

## Introduction

Myogenesis is the formation of muscular tissue during embryonic development, including the myoblast proliferation and differentiation into myocytes. In the early stage of embryonic development, multipotent mesenchymal stem cells (MSCs) were differentiated into myoblast by the regulation of specific transcription factors such as myoblast determination protein (MyoD), myogenin (MyoG), myogenic factor 5 (MYF5) and myogenic regulatory factor 4 (MRF4)^1–4^. Several genes and their protein products have been reported to be expressed and function in this process. Myocyte enhancer factors (MEFs) may promote myogenesis and serum response factor (SRF) may function in the process of myogenesis based on the expression of striated alpha-actin genes^5^. Myogenesis can also be regulated by steroids, being required for the expression of skeletal alpha-actin regulated by the androgen receptor^6^. In addition, some of epigenetic regulations were also identified to be essential in myogenesis, which may modify the transcriptional activity in many different biological processes of muscle development^7,8^. The regulation of microRNAs (miRNAs) was reported to play an increasingly important role in skeletal muscle cell proliferation and differentiation such as miR-34b, miR-17 and miR-20a^9,10^.

Peroxisome proliferator-activated receptor γ (PPARγ) is a member of the nuclear receptor superfamily whose basic function is to participate in regulating the process of adipocyte differentiation and glucose metabolism^11^. The transcriptional regulation is achieved by binding the peroxisome proliferator responsive elements that are upstream of the target genes promoters^12,13^. Indeed, it has been reported that PPARγ could play an important role in myogenic differentiation in skeletal muscle cells^14^. The effect of PPARγ in myogenic differentiation was just revealed by the phenotypic observations of cell differentiation and biochemical analysis of different myogenic marker genes expression. However, the underlying genetic mechanism of PPARγ regulating myogenic differentiation remains unknown.

To further elucidate the mechanisms of the effect of altered PPARγ expression in myogenic differentiation, we have performed the gene expression profiles of skeletal muscle differentiation between the wild-type and under the overexpression of PPARγ in C2C12 cells by RNA sequencing (RNA-seq). Our results would provide a comprehensive knowledge of genetic changes in response to PPARγ overexpression at a cellular level in transcriptomics.

## Materials and Method

### Cell Culture and Differentiation

Mouse skeletal muscle cell line C2C12 was purchased from Institute of Biochemistry and Cell Biology, Chinese Academy of Sciences (IBCB, CAS), Shanghai, China. The C2C12 cells were maintained in Dulbecco’s modified Eagle’s medium (DMEM, Sigma) supplemented with 10% fetal bovine serum (FBS, Gibco BRL, USA), MEM nonessential amino acids (Invitrogen), L-glutamine (Invitrogen), penicillin and streptomycin (Sigma), 0.01 mM β-mercaptoethanol (Invitrogen) and 1000 units/ml LIF(Millipore). The cells were cultured for 5 days that were considered mature with at least 95% conversion into the myogenic morphology. The control samples were collected from C2C12 cells on 0 day (0d), 1st day (1d), 3rd day (3d), and 5th day (5d).

### Plasmid construction and transfection

The coding sequence of PPARγ (NM _001127330.2) was resourced from the Genbank database, which was inserted into a PiggyBac vector (PB-IP) to form the sense constructs of PPARγ (PPARγ/+). Plasmids were expanded in *Escherichia coli* (strain DH5a). The recombinant plasmid was then transduced into C2C12 cells combined with 2 μg transposase using LTX (Invitrogen). After transfection, C2C12 cells were selected with 2 μg/ml puromycin for 5 days to obtain positive clones. The experimental samples were collected from the sense PPARγ C2C12 cells (PPARγ/+) on 0d, 1d, 3d and 5d.

### mRNA extraction and sequencing

Total RNA was extracted from the control samples of 0d and 5d (named as WT_0d and WT_5d) as well as from the experimental samples of 0d and 5d (named as PPARγ/+ _0d and PPARγ/+ _5d) using TRIzol. The replicate was triple. cDNA of each sample was synthesized from 2 μg total RNA using reverse transcriptase (Takara) according to the manufacturer’s instructions. The preparation of the cDNA library and the RNA sequencing was performed by Genergy Biotechnology (Shanghai, China). The cDNA originating from the RNA fragments were paired and sequenced using the high throughput sequencing platform of Illumina HiSeq. 3000, and 6 G reads per sample were obtained on average. Our sequencing data has been submitted to the Gene Expression Omnibus (GEO) database and assigned the accession number as GSE99399.

### Analysis of RNA-Seq data

Analysis of our RNA-Seq data was performed based on the regular protocol as follows^15^. The Trim Galore software was used to complete 3′ end dynamic removal of linker sequence fragments and low mass fragments of sequencing data. Analysis of quality control of the preprocessing data was performed by FastQC software. For each sample, the STAR software was used for sequence alignment between the preprocessing sequence and reference genome sequence of mice downloaded from the Ensembl database (Mus_musculus.GRCm38.83, ftp://ftp.ensembl.org/pub/release-83/gtf/mus_musculus/Mus_musculus.GRCm38.83.chr.gtf.gz). Transcript assembly of RNA-seq data from each sample was performed by the Cufflinks software. Based on the Cufflinks treated reads, the software of Cuffcompare was used to parsimoniously merge the assembled transfrags.

The HTSeq software was used to count the original sequence of known genes for all samples, the expression of the known gene was calculated by Fragments Per Kilobase of transcript per Million fragments mapped (FPKM). The clustering of each sample was performed by Principal Component Analysis (PCA) as well as calculating the Pearson correlation. DESeq. 2 was used to perform the analysis of differentially expressed genes (DEG). The cutoffs of DEG approach were chosen as the p value < = 0.05 and the Fold Change (FC) value > = 2. Hierarchical clustering was performed on the DEGs according to the distance metric of Pearson correlation and the average linkage. Functional annotations of the DEGs including GO (Gene Ontology) and KEGG (Kyoto Encyclopedia of Genes and Genomes) pathways enrichment were performed using the approach of gene set enrichment analysis (GSEA)^16,17^. Cytoscape was used to reconstruct the regulatory networks.

### Quantitative polymerase chain reaction (qPCR) for target genes

Primers of target genes were designed using Primer 3.0 input software. QPCR was performed with TransStart Tip Green qPCR SuperMix (Takara). The expression level of each transcript was normalized with glyceraldehyde-3- phosphate dehydrogenase (GAPDH) and analyzed using the 2−ΔΔCt method.

### Immunofluorescence staining

Cells were fixed in 4% paraformaldehyde for 20 minutes and incubated at 37 °C in blocking buffer (PBS containing 5%BSA and 0.2% Triton X-100). Cells were incubated in the presence of primary antibodies at 4 °C overnight and then washed three times in 1 × PBS. Cells were then incubated with Alexa Fluor 594 (Invitrogen, 1:1000) secondary antibody for 1 h at 37 °C. Nuclei were stained with Hoechst (Invitrogen, 1:5000). The primary antibodies and dilutions used for MyoD (sc-32758; Santa Cruz Biotechnology, 1:200).

## Results and Discussion

### Overexpression of PPARγ inhibits myogenic differentiation in C2C12

**Figure 1:**
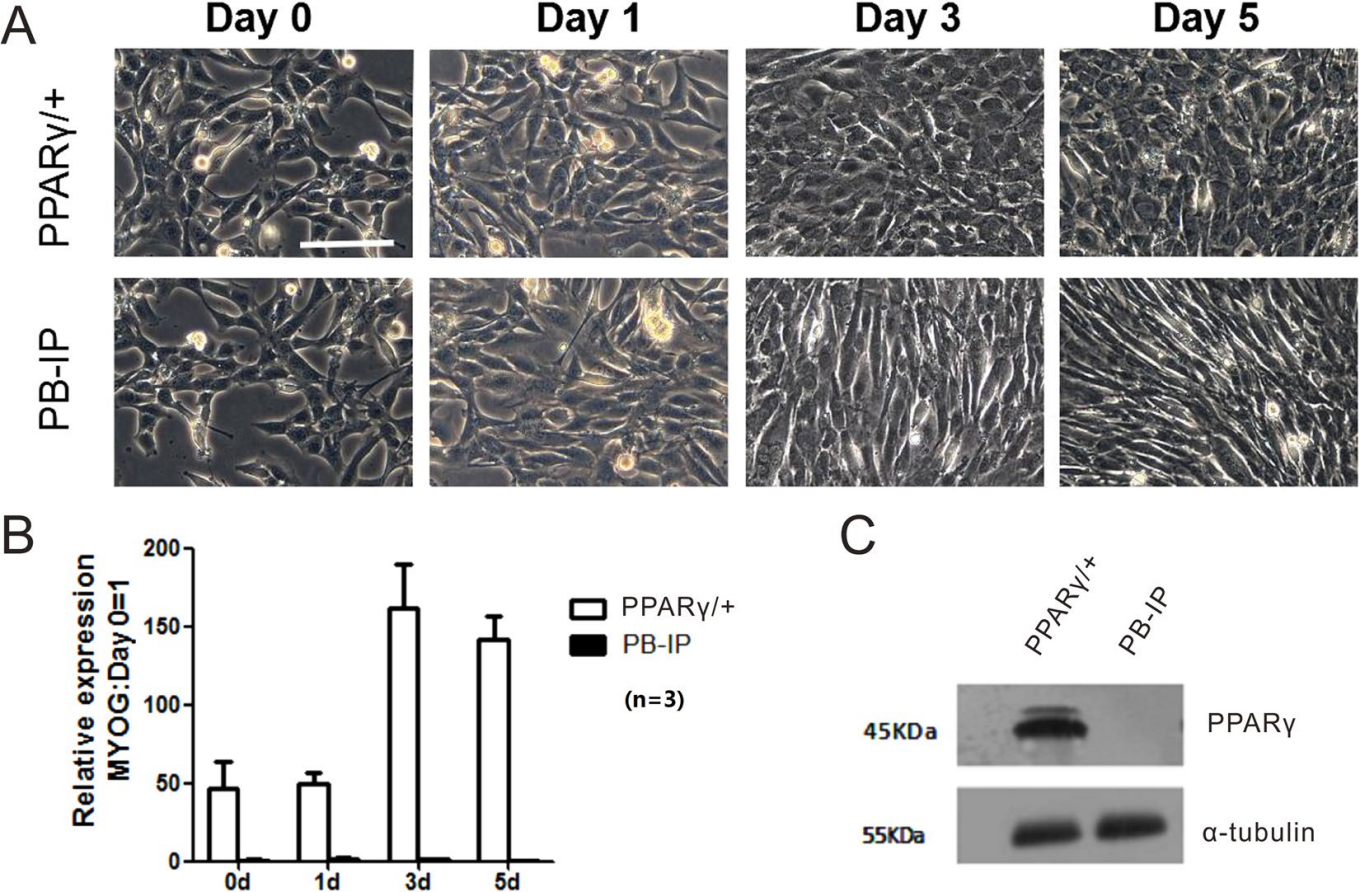
The reconstruction of PPARγ overexpression in the development of C2C12. (**A**) The overexpression of PPARγ may inhibit myogenic differentiation in C2C12 myoblasts. During the differentiation of C2C12 from 0d to 5d, a marked reduction in its ability to form myotubes was found in C2C12 cells overexpressing PPARγ. Scale bar:100 μm. (**B**) The stable C2C12 cell lines under the overexpression of PPARγ by transfection of recombinant plasmid have been established. Compared with the wild-type (WT) PB-IP group, the expression of PPARγ gene was detected to be significantly higher in the group of PPARγ/+ by qRT-PCR. The number of replicates was three (n = 3). (**C**) The protein of PPARγ was detected to be significantly higher in the group of PPARγ/+ by Western blot.

According to the observation of cellular characteristics, the overexpression of PPARγ may inhibit myogenic differentiation in C2C12 skeletal muscle cells. During the development of C2C12 from 0d to 5d, a marked reduction in its ability to form myotubes was found in C2C12 cells overexpressing PPARγ (Fig. 1A). We have established the stable C2C12 cell lines under the overexpression of PPARγ by transfection of recombinant plasmid. Compared with the wild-type (WT) PB-IP group, the expression of PPARγ was detected to be significantly higher in the group of PPARγ/+ by qRT-PCR as well as Western blot during the cell differentiation, especially on the last two periods including 3d and 5d (Fig. 1B and C). The results were consistent with previous reports^14^.

**Figure 2:**
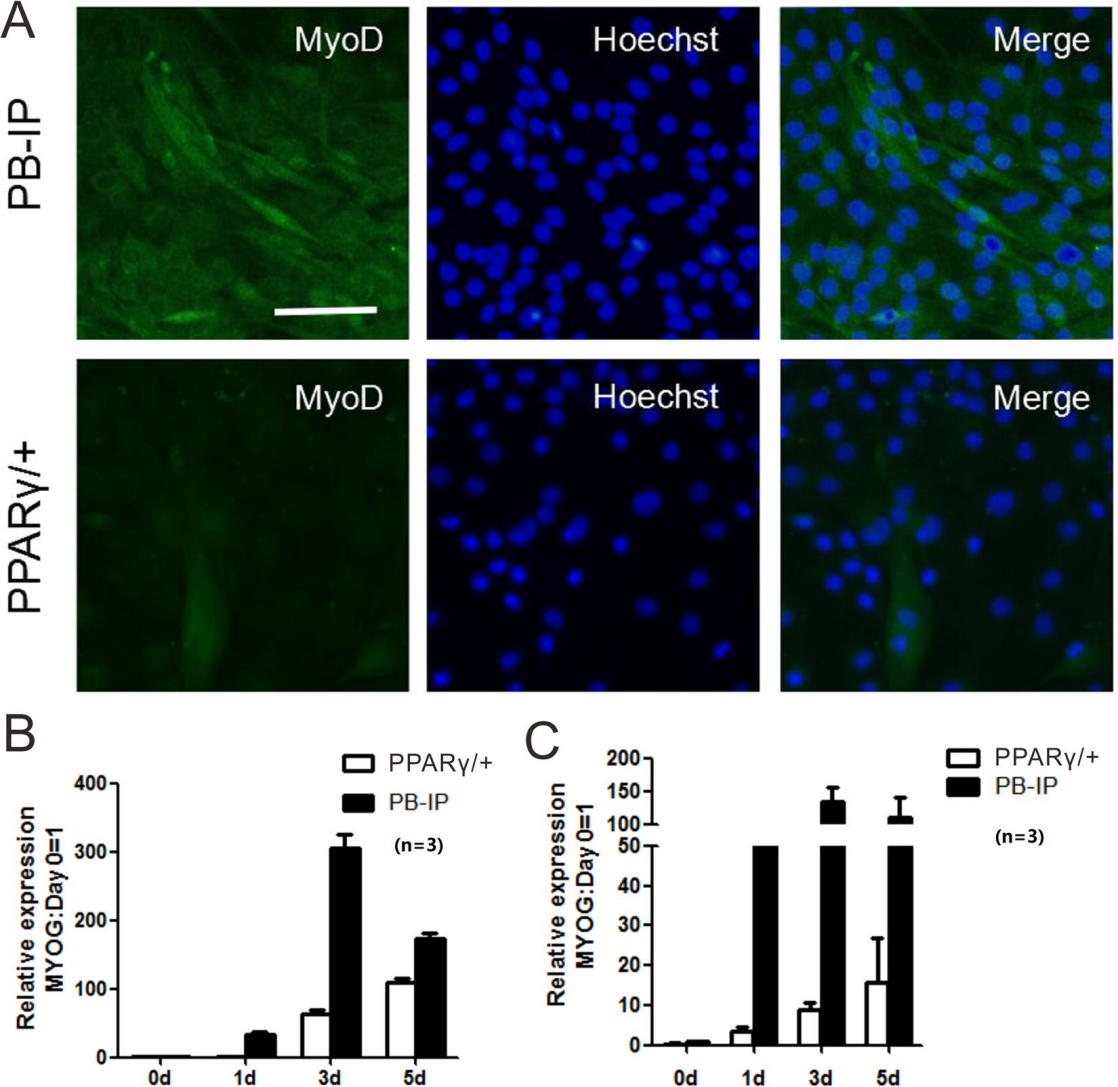
Immunofluorescence analysis and Western blot of myogenic marker genes under PPARγ overexpression in C2C12 cells after 5 days of differentiation. (**A**) Based on the immunofluorescence analysis, MyoD expression was affected by overexpression of PPARγ in C2C12. Scale bar:100 μm. (**B**) Lower level of MyoD expression was detected in PPARγ/+ cells compared with WT cells by the test of Western blot. The number of replicates was three (n = 3). (**C**) Lower level of myogenin expression was detected in PPARγ/+ cells compared with WT cells by the test of Western blot.

To further validate the observed cellular phenomenon, biochemical investigation of two well-known muscle differentiation markers myogenin and MyoD was performed to determine their expression levels and creatine kinase activity. In this study, both myogenin and MyoD expressions were affected by overexpression of PPARγ in C2C12 based on the immunofluorescence analysis (Fig. 2A). Correspondingly, lower level of both myogenin and MyoD expressions were detected in PPARγ/+ cells compared with WT cells by the test of Western blot (Fig. 2B and C).

According to both cellular phenotypes and molecular characters, it is suggested that the critical roles of PPARγ in myoblasts differentiation may be inhibited by the overexpression of PPARγ.

### The overview of RNA sequencing data

**Figure 3:**
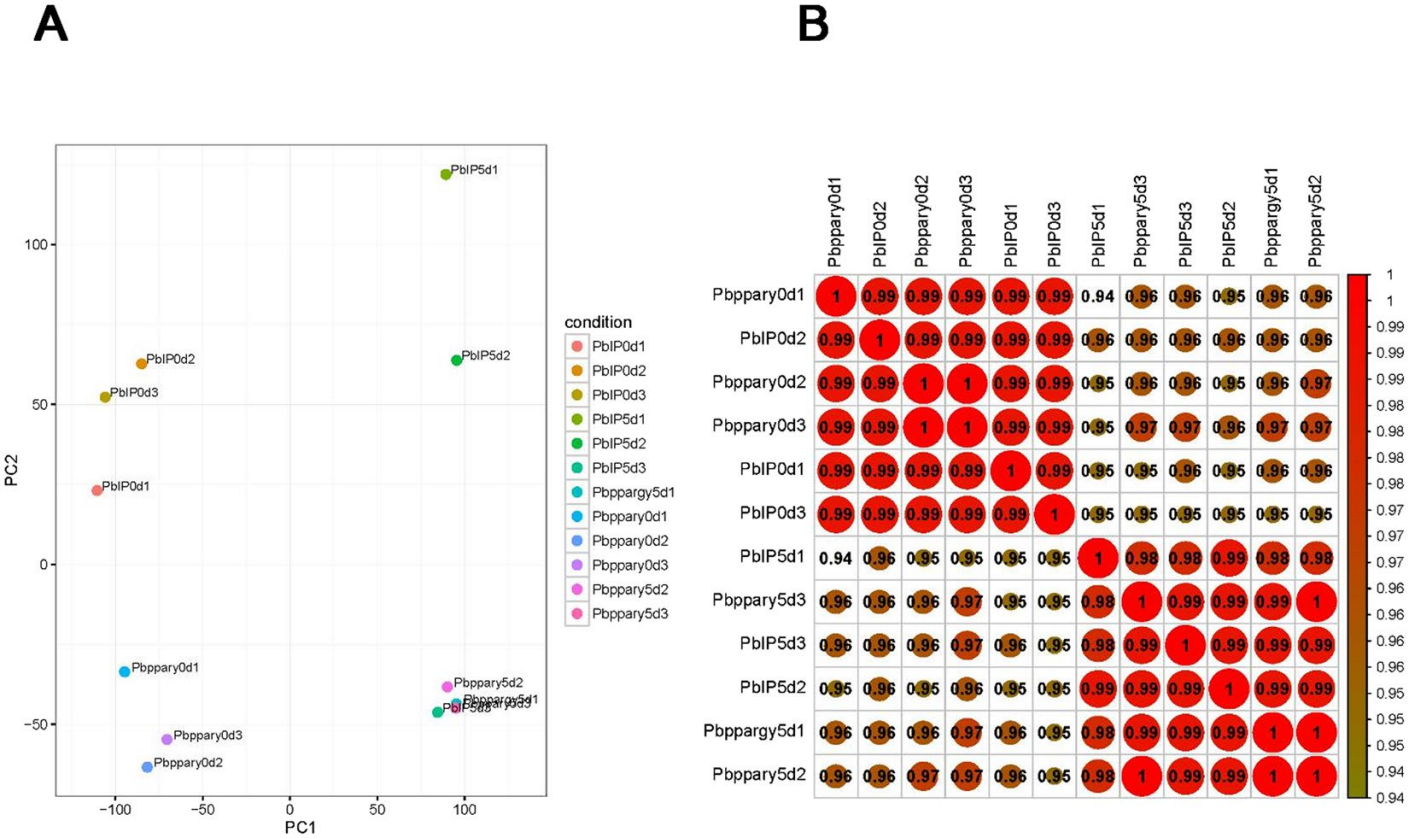
Data quality of RNA-seq. (**A**) Principal component analysis (PCA) results showed that the replicate samples could be clustered in the same groups, including the group of WT on 0d, WT on 5d, PPARγ/+ on 0d and PPARγ/+ on 5d. (**B**) The correlation analysis result was the same as the PCA result.

In order to further study the molecular mechanisms of PPARγ regulating myogenic differentiation, we have performed transcript profiling of WT cells as well as PPARγ/+ cells on both 0d and 5d by RNA-seq. Totally there were 12 sequencing samples, each group has triple biological replicates. Based on data quality control and preprocessing, clean reads and the ratios of cleans reads in raw reads were obtained for each sample. Q20 and Q30, the percentage of bases with Phred values >20 and >30, were also evaluated respectively. The details of RNA sequencing data were shown in Table 1. After the sequencing reads alignment to the mouse reference genome, the mapping rate of each sample exceeded 97.5% was obtained (Table S1). The PAC results and the correlation analysis showed that internal consistency of replicate samples in the same group (including four groups of WT_0d, WT_5d, PPARγ/+ _0d and PPARγ/+ _5d) was good (Fig. 3A and B). All of these indicate that our RNA sequencing data were of high quality with sufficient sequencing depth for the next differential expression analysis.

**Table 1:** The summary of raw RNA sequencing data.

Sample	Raw reads	Clean reads	Clean reads %	Q20%	Q30%
PbIP0d1(WT_0d_1)	40596792	39745948	97.90%	96.49%	91.11%
PbIP0d2(WT_0d_2)	52272774	51608354	98.73%	97.13%	92.43%
PbIP0d3(WT_0d_3)	48147658	47390122	98.43%	96.83%	91.78%
PbIP5d1(WT_5d_1)	52875822	52050588	98.44%	96.82%	91.77%
PbIP5d2(WT_5d_2)	40112764	39503346	98.48%	96.84%	91.79%
PbIP5d3(WT_5d_3)	47268810	46603152	98.59%	96.93%	92%
Pbppary0d1(PPARγ/+ _0d_1)	50606028	49842620	98.49%	96.89%	91.93%
Pbppary0d2(PPARγ/+ _0d_2)	53914618	53276270	98.82%	97.17%	92.51%
Pbppary0d3(PPARγ/+ _0d_3)	45421192	44669694	98.35%	96.79%	91.73%
Pbppary5d1(PPARγ/+ _5d_1)	43011810	42382296	98.54%	96.91%	91.97%
Pbppary5d2(PPARγ/+ _5d_2)	44606890	44006710	98.65%	96.98%	92.08%
Pbppary5d3(PPARγ/+ _5d_3)	38524764	38001320	98.64%	96.97%	92.06%

It showed the summary of RNA sequencing data of 12 samples, including raw reads, clean reads, and percentage of clean reads as well as Q20% and Q30% (the percentage of bases with Phred values > 20 and > 30).

### Identification of differentially expressed genes related to PPARγ regulation

**Figure 4:**
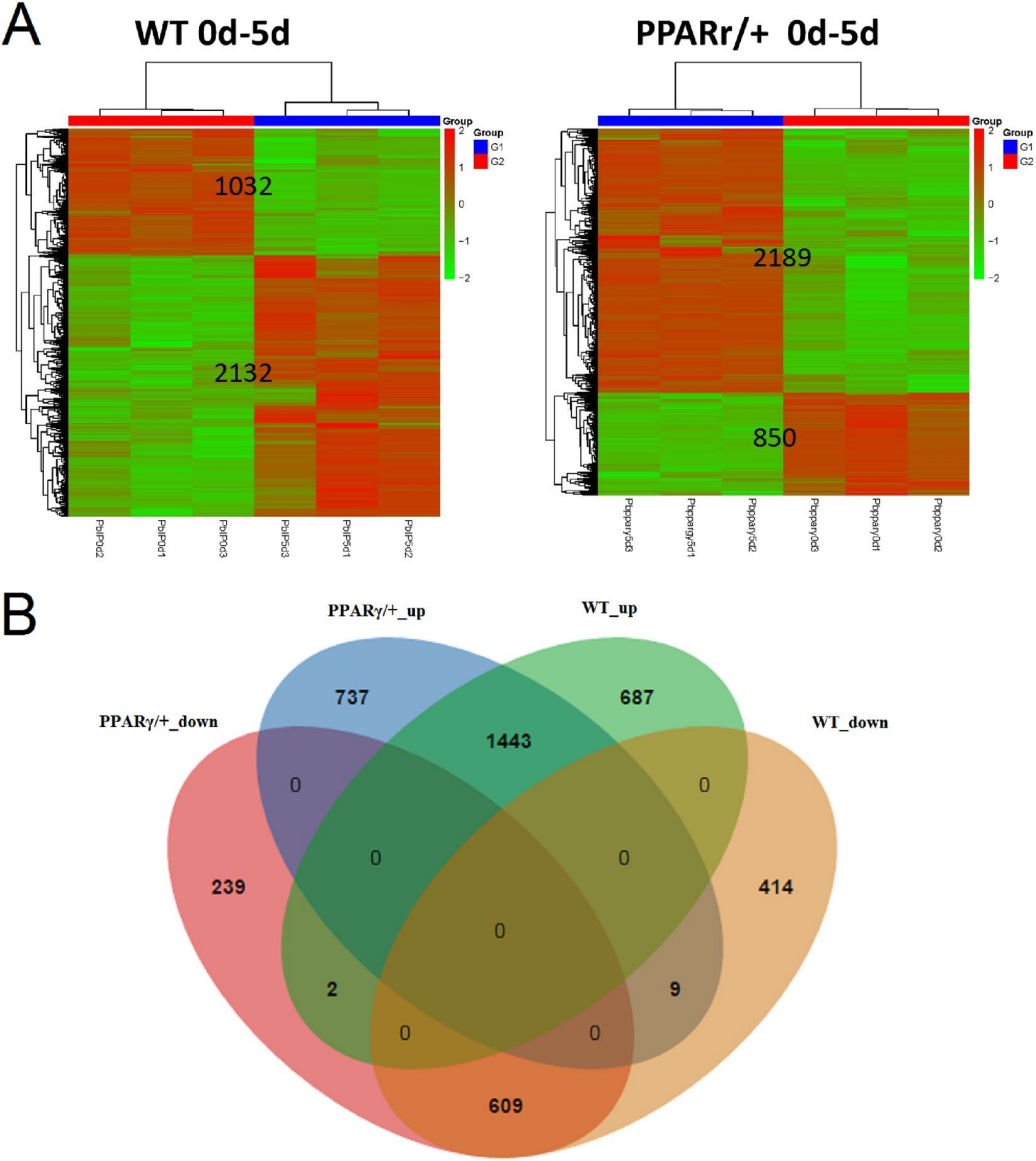
The comparison of differentially expressed genes between wild-type cells and PPARγ/+ cells. (**A**) The heatmap showed expression patterns of significantly regulated genes, respectively for wild-type (WT) and PPARγ/+ cells. According to differentially expressed genes (DEG) analysis, 3164 significantly dysregulated genes with FC more than 2 and p less than 0.05, including 2132 up-regulated and 1032 down-regulated genes were identified in WT cells from 0d to 5d. In contrast, 3039 significantly dysregulated genes including 2189 up-regulated and 850 down-regulated genes were identified in PPARγ/+ cells from 0d to 5d. (**B**) The Venn diagram showed the common genes in both of WT cells and PPARγ/+ cells. There were 1443 common up-regulated genes and 609 common down-regulated genes identified in both of two different cells.

According to the transcriptome assembly, there were totally 27138 annotated transcripts in our mRNA-seq data. By calculating the differential expression and analyzing the significance of each gene between 0d and 5d, 3164 significantly dysregulated genes with FC more than 2 and p value less than 0.05, including 2132 up-regulated and 1032 down-regulated genes were identified in WT cells from 0d to 5d. In contrast, 3039 significantly dysregulated genes including 2189 up-regulated and 850 down-regulated genes were identified in PPARγ/+ cells from 0d to 5d. The expression patterns of significantly regulated genes were shown in Fig. 4A, and the details of each gene were indicated in Table S2. To indentify the common genes in both of WT cells and PPARγ/+ cells, we have compared the significantly dysregulated genes in each group. As a result, 1443 common up-regulated genes and 609 common down-regulated genes were identified in both of two different cells, which were shown in Fig. 4B and Table S3.

**Figure 5:**
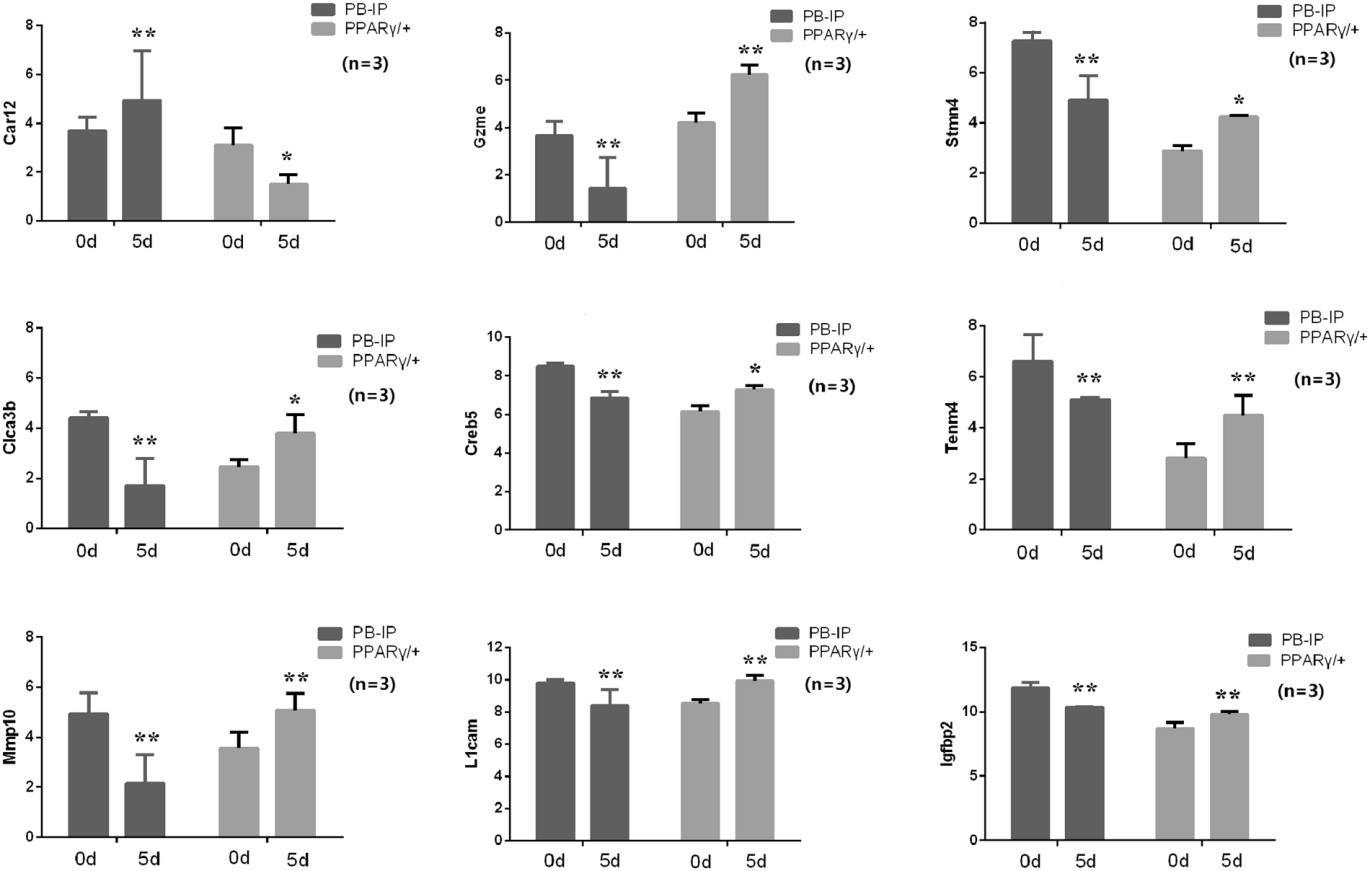
The 9 specific genes with diverse regulatory patterns in response to PPARγ overexpression. There were 9 specific genes with diverse regulatory patterns in response to PPARγ overexpression, which were validated by qRT-PCR. Among them, 8 genes including *Stmn4*, *L1cam*, *Tenm4*, *Clca3b*, *Gzme*, *Mmp10*, *Igfbp2* and *Creb5* were significantly down-regulated in WT cells but significantly up-regulated in PPARγ/+ cells from 0d to 5d. Only one gene *Car12* was significantly up-regulated in WT cells but significantly down-regulated in PPARγ/+ cells from 0d to 5d. The number of replicates was three (n = 3). ** indicates most significant level, * indicates significant level.

Furthermore, 11 specific genes with diverse regulatory patterns were identified, the details of which were shown in Table 2 and Fig. 5. Among them, 9 genes including *Stmn4*, *L1cam*, *Tenm4*, *Clca3b*, *Gzme*, *Mmp10*, *Igfbp2*, *Creb5* and *Slc14a1* were significantly down-regulated in WT cells but significantly up-regulated in PPARγ/+ cells from 0d to 5d. In zebrafish study, the functional analysis of stathmin-like 4 (*Stmn4*) had shown its essential roles in the maintenance of neural progenitor cells, whose expression was revealed to be enriched in the diencephalon *in situ* hybridization analysis^18,19^. STMN4 gene has been also reported to be involved in neuroblastoma cell differentiation^20^. L1 cell adhesion molecule (*L1cam*) may also play important roles in nervous system development, regeneration and neurodegeneration as well as in tumorigenesis^21^. In *Caenorhabditis elegans* (*C. elegans*), SAX-7/L1CAM was reported to function in localized myosin accumulation and actomyosin contractility during gastrulation^22^. In mice, TENM4 is a regulator of axon guidance and central myelination, which is absolutely necessary for mesoderm induction^23,24^. Ca(+)-activated Cl(−) channel (CLCA) proteins function in the biological process of inflammation and chloride transport^25^. A family of serine proteases granzymes whose gene expressions present cell specificity may function in the cytolytic potential of T effector cells^26,27^. During early embryogenesis, matrix metalloproteinase 10 (*Mmp10*) may play an important role in the maintenance of vascular integrity by the association with histone deacetylase 7 (HDAC7)^28^. Altered expression level of insulin-like growth factor binding protein-2 (IGFBP2) has been identified in skeletal muscle of bovine fetuses at early gestation from embryos produced *in vivo* or *in vitro* ^29^. cAMP-responsive element binding protein 5 (CREB5) targeted by miR-449a is able to influence hepatitis B virus (HBV) replication^30^. In *C. elegans*, CREB5 functions with other transcriptional regulators such as activating transcription factor (ATF) family ATF2/ATF7 in the control of innate immunity^31^. The solute carrier family 14 (urea transporter), member 1 (SLC14A1) gene has been identified as one of essential biomarkers of bladder cancer by genome-wide association studies (GWAS) and RNA-seq^32^.

**Table 2:** The 11 specific genes with diverse regulatory patterns in response to PPARγ overexpression.

Ensemble ID	Symbol	log(FC) in PPARγ/+	P value in PPARγ/+	log(FC) in WT	P value in WT
ENSMUSG00000031391	*L1cam*	1.40	9.15E-05	−1.46	2.48E-03
ENSMUSG00000048078	*Tenm4*	1.68	5.62E-03	−1.50	2.51E-03
ENSMUSG00000039323	*Igfbp2*	1.11	2.44E-03	−1.52	4.32E-05
ENSMUSG00000053007	*Creb5*	1.13	1.65E-02	−1.61	9.65E-04
ENSMUSG00000022156	*Gzme*	2.04	2.09E-04	−2.22	8.58E-03
ENSMUSG00000022044	*Stmn4*	1.37	3.50E-02	−2.33	1.21E-04
ENSMUSG00000037033	*Clca3b*	1.32	3.59E-02	−2.72	1.45E-03
ENSMUSG00000047562	*Mmp10*	1.52	8.99E-03	−2.78	3.36E-04
ENSMUSG00000059336	*Slc14a1*	1.98	1.06E-02	−3.03	9.48E-05
ENSMUSG00000032373	*Car12*	−1.59	1.80E-02	1.25	3.95E-03
ENSMUSG00000048721	*Fndc9*	−1.62	4.25E-02	1.58	2.98E-02

It showed the fold change (FC) and significance level of p value for 11 specific genes with diverse regulatory patterns in response to PPARγ overexpression, including 9 genes *Stmn4*, *L1cam*, *Tenm4*, *Clca3b*, *Gzme*, *Mmp10*,* Igfbp2*, *Creb5* and *Slc14a1* were significantly down-regulated in WT cells but significantly up-regulated in PPARγ/+ cells from 0d to 5d. On the contrary, only 2 genes including *Car12* and *Fndc9* were significantly up-regulated in WT cells but significantly down-regulated in PPARγ/+ cells from 0d to 5d.

On the contrary, only 2 genes including *Car12* and *Fndc9* were significantly up-regulated in WT cells but significantly down-regulated in PPARγ/+ cells from 0d to 5d. As one of membrane-bound isozymes, carbonic anhydrase 12 (*Car12*) was expressed at higher levels during mammalian inner ear development^33^. CAR12 expression could be upregulated by estrogen, which may reveal a novel regulatory mechanism underlying cyclic changes in mouse uterine bicarbonate secretion^34^. Although less functional information has been known for fibronectin type III domain containing 9 (*Fndc9*), another family gene Fndc5 in skeletal muscle was well studied on its association with endurance exercise not only in rats but also in pigs^35,36^.

### Functional enrichment in response to PPARγ overexpression

**Figure 6:**
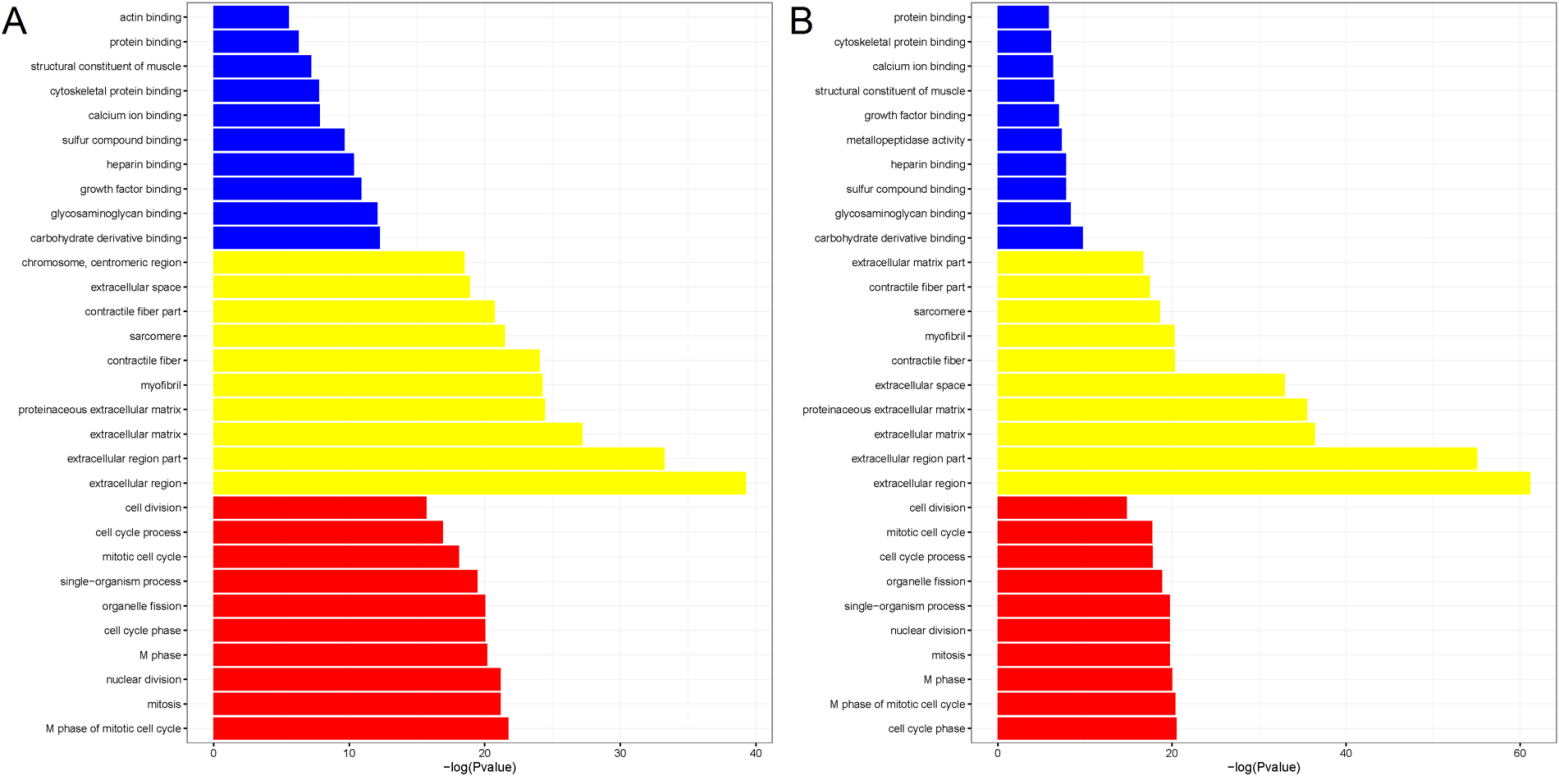
The comparison of GO enrichment. It showed the top 10 significantly enriched GO terms including molecular function (MF) in blue color, cellular component (CC) in red color and biological process (BP) in yellow color, respectively for WT (**A**) and PPARγ/+ cells (**B**). The X-axis represents the significance level as –log(p value), the Y-axis represents each name of GO terms.

According to functional annotations of the DEGs in response to the overexpression of PPARγ, several GO terms including molecular function (MF), cellular component (CC) and biological process (BP) were significantly enriched (shown in Fig. 6 and Supplemental Figs 1 and 2). The top 10 terms of CC and BP were similar before and after the overexpression of PPARγ (red and yellow parts in Fig. 6). For the top 10 terms of MF in the wild-type cells from 0d to 5d, most of them were enriched in the terms of binding such as carbohydrate derivative binding and cytoskeletal protein binding as well as the term of structural constituent of muscle (blue part in Fig. 6A). Besides, the term of metallopeptidase activity was enriched exclusively in the PPARγ/+ cells from 0d to 5d (blue part in Fig. 6B). In the previous study of anti-inflammatory activity role of PPARγ in skeletal muscle, one of inflammatory molecules matrix metalloproteinase-2 (MMP-2) was reported to be positively co-expressed with PPARγ in muscles of the acute exercise group, but it was dramatically reduced by PPARγ overexpression^37^.

**Figure 7:**
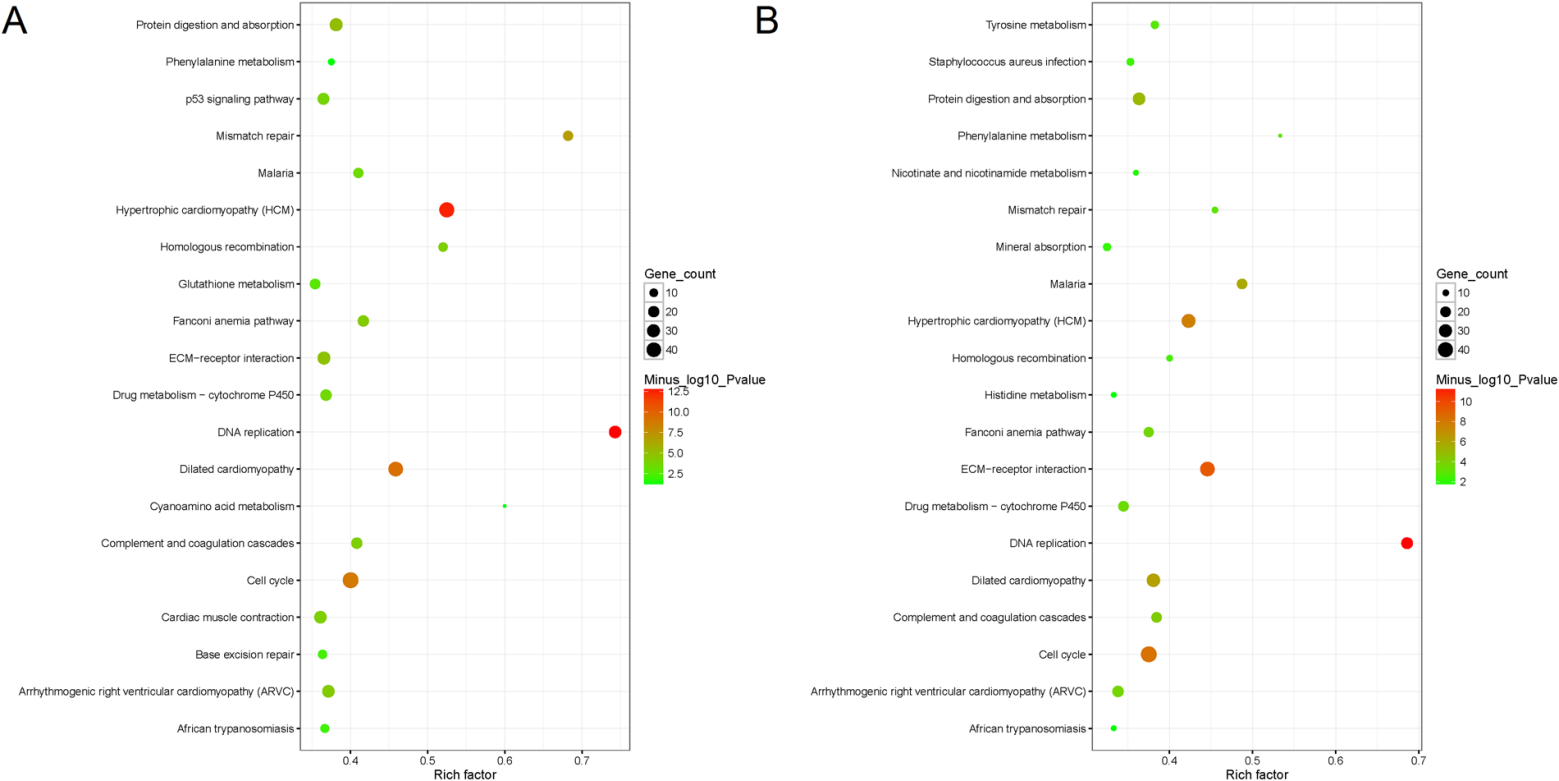
The comparison of pathway enrichment. It showed the top 20 significantly enriched KEGG pathways, respectively for WT (**A**) and PPARγ/+ cells (**B**). The X-axis represents the rich factor of each pathways, the Y-axis represents each name of KEGG pathways. Round size represents the counts of genes involved in each significant pathway. Color type represents the significance level as –log(p value), red represents most significant and green represents less significant.

Based on the enrichment analysis of KEGG pathways shown in Fig. 7, the top pathway with the highest rich factor was DNA replication in both of two diverse cell types, which is the basic process of genetics. However, significantly enriched p53 signaling pathway in the wild-type cells no longer existed in the PPARγ/+ cells, which revealed that the activity of p53 signaling pathway would be inhibited by the overexpression of PPARγ in muscle cell development. The vascular smooth muscle cell apoptosis may be induced by one of activators of PPARγ troglitazone, the effect of which is thought to be caused primarily by activation of the p53 pathway^38^. Moreover, some of specific pathways such as nicotinate and nicotinamide metabolism as well as histidine metabolism were identified exclusively in the PPARγ/+ cells. Niacin-induced PPARγ transcriptional activity has been well studied in macrophages via HM74 and HM74a-mediated induction of prostaglandin synthesis pathways^39^. A single amino acid histidine in PPARγ has been reported to determine the ligand binding selectivity by a high-resolution cocrystal structural study^40^.

### Regulatory network reconstruction in response to PPARγ overexpression

**Figure 8:**
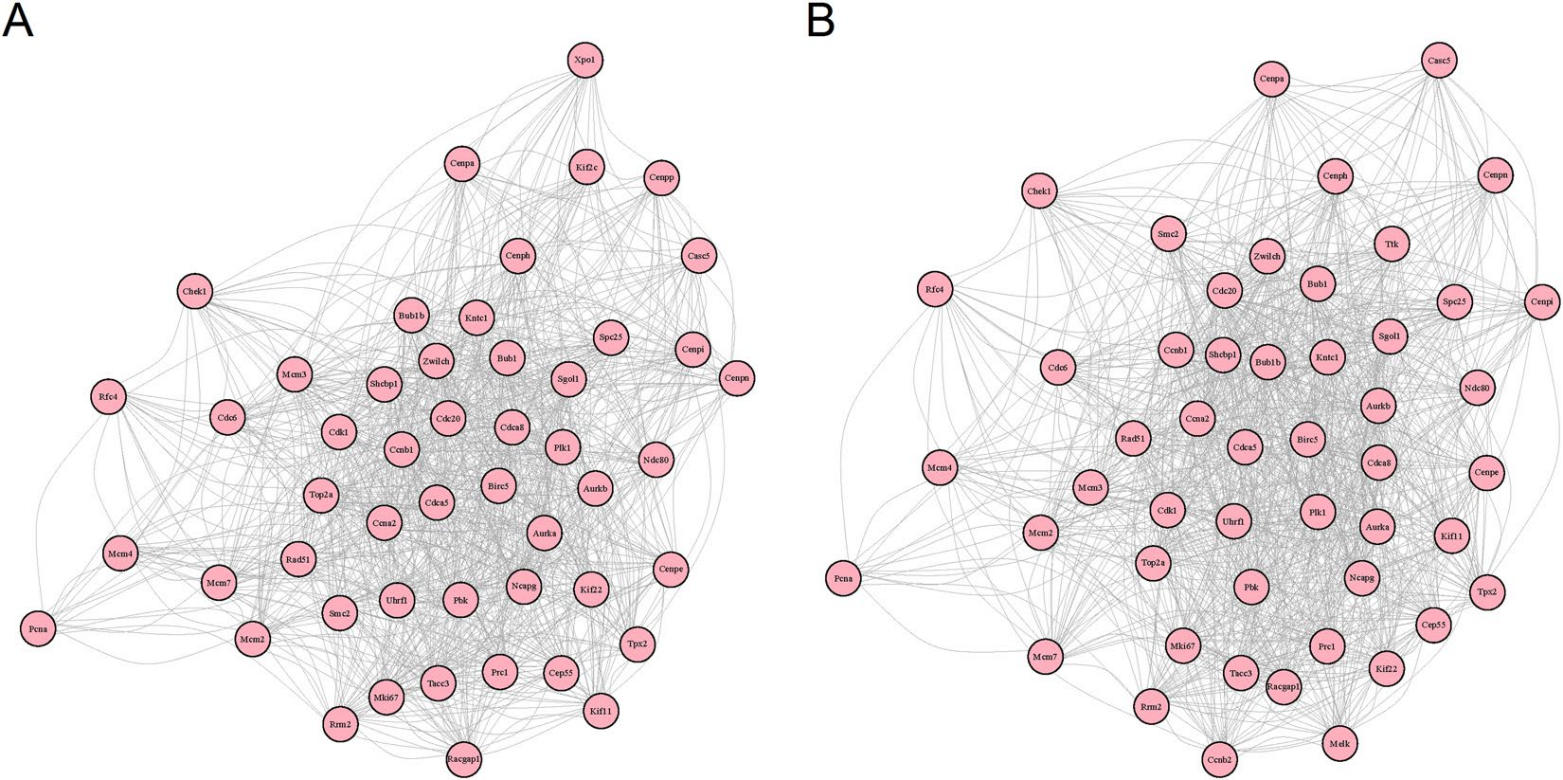
Reconstruction of regulatory networks in response to PPARγ overexpression. The regulatory networks in response to PPARγ overexpression have been reconstructed, respectively for WT (**A**) and PPARγ/+ cells (**B**). For these two cell types, there were both 50 genetic factors involved in each network, most of which were common genes annotated in several gene families such as the family of *Aurks*, *Cdcs*, *Cenps* and *Mcms*. 3 specific genes including *Cenpp*, *Kif2c* and *Xpo1* were involved in the network for wild-type cells, and other 3 specific genes including *Ccnb2*, *Melk* and *Ttk* were involved in the network for PPARγ/+ transfected cells.

To further demonstrate the genetic mechanism of myogenic differentiation in response to PPARγ overexpression, the gene regulatory networks have been reconstructed for the PPARγ/+ transfected cells and the wild-type cells, respectively (Fig. 8). For these two cell types, there were both 50 genetic factors involved in each network, most of which were common genes annotated in several gene families such as the family of *Aurks*, *Cdcs*, *Cenps* and* Mcms*. 3 specific genes including *Cenpp*, *Kif2c* and *Xpo1* were involved in the network for wild-type cells, and other 3 specific genes including *Ccnb2*, *Melk* and *Ttk* were involved in the network for PPARγ/+ transfected cells. One of centromere proteins (CENPs) family Mitosin/CENP-F is a conserved kinetochore protein subjected to cytoplasmic dynein-mediated poleward transport, which may function in mitosis, transcriptional control, and differentiation^41,42^. In vascular smooth muscle cells, the nuclear autoantigen CENP-B can stimulate the trans-activation of the epidermal growth factor receptor (EGFR) via chemokine receptor 3 (CCR3) and display cytokine-like activities as well^43,44^. Mitotic centromere-associated kinase (MCAK/Kif2C) functions not only in chromosome movement and segregation with ATP-dependent microtubule depolymerase activity, but also in the regulation of cellular senescence through a p53-dependent pathway and tissue/organism aging and protection of cellular transformation as well^45^. The nucleocytoplasmic transport-related gene of exportin 1 (XPO1) has been reported to play a critical role in the pathologies of ischemic and dilated cardiomyopathies^46^. The expressions of cyclin B2 (*Ccnb2*) and PPARγ were reported to be co-regulated in the study of the effects of myostatin on the proliferation and differentiation of 3T3-L1 preadipocytes, which is a critical negative regulator of skeletal muscle development^47^. Maternal embryonic leucine zipper kinase (MELK), a modulator of intracellular signaling, may function in affecting various cellular and biological processes, including cell cycle control, embryonic development, and multiple cancers^48^. In *Drosophila melanogaster*, the pleiotropic transcriptional repressor Tramtrack69, one of TTK protein kinases, has been identified a new functional role in stabilizing cell fate during myogenesis^49^.

## Conclusion

The role of the PPARγ in myogenesis draws little attention before. In the present study, we have reconstructed the overexpression of PPARγ in C2C12 cells and investigated subsequent alteration of transcriptome. We have confirmed that the formation of myotubes as well as the expression of muscle-specific myogenic genes such as *MyoD* and *MyoG* may be inhibited by PPARγ overexpression. Multiple genes and pathways were significantly involved in this process, including 11 genes such as *Fndc9* and *Slc14a1* with fundamental change of regulation modes, 9 genes of which were validated by the data of qRT-PCR. It is somewhat helpful to reveal the complex network of myogenesis, even though PPARγ is not a dominant factor involved in it.

## Electronic supplementary material

Supplementary Information

Table S1

Table S2

Table S3
