# Supplementary material for: A transcriptomic study of myogenic differentiation under the overexpression of PPARγ by RNA-Seq
Source: Sci Rep. 2017 Nov 10;7:15308. doi: 10.1038/s41598-017-14275-2 (PMC5681552; doi:10.1038/s41598-017-14275-2)
Supplement: Supplementary file 1 — Supplementary Information [file 41598_2017_14275_MOESM1_ESM.pdf]

A transcriptomic study of myogenic differentiation under the overexpression of PPARγ by RNA-Seq

Kan He[1,2,3#*], Guoying Wu[3#], Wen-Xing Li[4,5,#], Daogang Guan[2#], Wenwen Lv[6], Mengting Gong[3], Shoudong Ye[1,3], Aiping Lu[2*]

[1] Department of Biostatistics, School of Life Sciences, Anhui University, Hefei 230601, Anhui, China

[2] School of Chinese Medicine, Hong Kong Baptist University, 7 Baptist University Road, Kowloon Tong, Hong Kong, China

[3] Center for Stem Cell and Translational Medicine, School of Life Sciences, Anhui University, Hefei 230601, Anhui, China

[4] State Key Laboratory of Genetic Resources and Evolution, Kunming Institute of Zoology, Chinese Academy of Sciences, Kunming 650223, Yunnan, China

[5] Kunming College of Life Science, University of Chinese Academy of Sciences, Kunming 650204, Yunnan, China

[6] Hongqiao International Institute of Medicine, Shanghai Tongren Hospital/Faculty of Public Health, School of Medicine, Shanghai Jiao Tong University, Shanghai 200025, China

[#] These authors contributed equally to this work.

*Corresponding authors:

Kan He (hekan_803@163.com)

Aiping Lu (aipinglu@hkbu.edu.hk)

Tel: +86 0551 63861819

Fax: +86 0551 63861819

Supplementary files

Figure S1. GO tree in WT cells

It showed the tree of enriched GO terms in WT cells, including molecular function (MF) in blue color, cellular component (CC) in red color and biological process (BP) in yellow color.

Figure S2. GO tree in PPARγ/+ cells

It showed the tree of enriched GO terms in PPARγ/+ cells, including molecular function (MF) in blue color, cellular component (CC) in red color and biological process (BP) in yellow color.

Table S1. The details of RNA sequencing data for each sample

It showed the details of RNA sequencing data of 12 samples, including total reads, total mapped reads, mapped ratio(%), multiple mapped, unique mapped, Read-1, Read-2, Reads map to '+', Reads map to '-', Non-Splice reads, Splice reads, Reads Proper pair.

Table S2. The details of significantly regulated genes in each group

It showed the details of significantly regulated genes from 0d to 5d respectively for WT and PPARγ/+ cells, including the information of normalized expression values, average expression values, log2 of fold change (FC), significance level of p value as well as false discover rate (FDR) for each significant genes.

Table S3. The details of commonly dysregulated genes

It showed the Ensembl IDs of commonly dysregulated genes from 0d to 5d in both WT and PPARγ/+ cells.

Figure S1

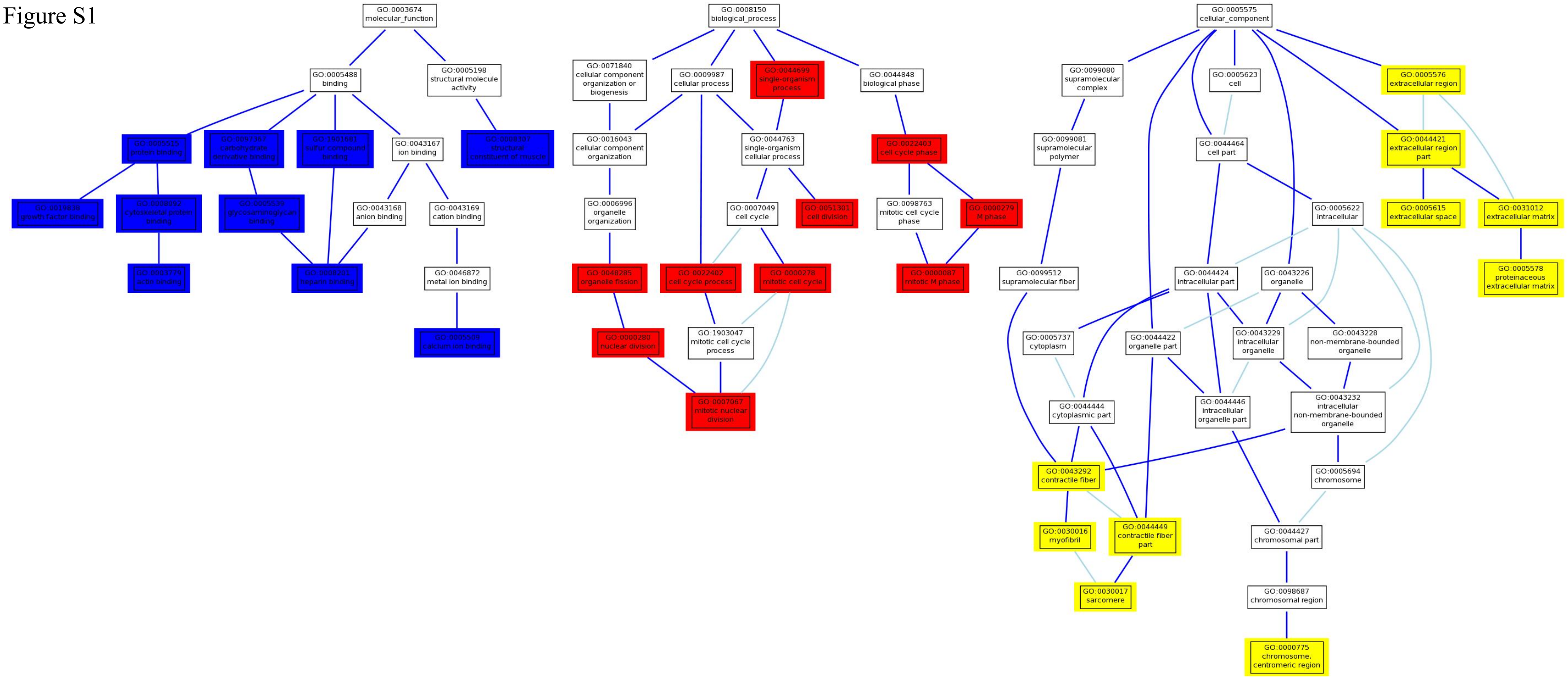

GO:0003674
molecular_function
GO:0005488
binding
GO:0005198
structural molecule activity
GO:0005515
protein binding
GO:0097367
carbohydrate derivative binding
GO:1901681
sulfur compound binding
GO:0043167
ion binding
GO:0008307
structural constituent of muscle
GO:0019838
growth factor binding
GO:0008092
cytoskeletal protein binding
GO:0005539
glycosaminoglycan binding
GO:0043168
anion binding
GO:0043169
cation binding
GO:0003779
actin binding
GO:0008201
heparin binding
GO:0046872
metal ion binding
GO:0005509
calcium ion binding
GO:0008150
biological_process
GO:0071840
cellular component organization or biogenesis
GO:0009987
cellular process
GO:0044699
single-organism process
GO:0044848
biological phase
GO:0016043
cellular component organization
GO:0044763
single-organism cellular process
GO:0022403
cell cycle phase
GO:0006996
organelle organization
GO:0007049
cell cycle
GO:0051301
cell division
GO:0098763
mitotic cell cycle phase
GO:0000279
M phase
GO:0048285
organelle fission
GO:0022402
cell cycle process
GO:0000278
mitotic cell cycle
GO:0000087
mitotic M phase
GO:0000280
nuclear division
GO:1903047
mitotic cell cycle process
GO:0007067
mitotic nuclear division
GO:0005575
cellular_component
GO:0099080
supramolecular complex
GO:0005623
cell
GO:0005576
extracellular region
GO:0099081
supramolecular polymer
GO:0044464
cell part
GO:0044421
extracellular region part
GO:0005622
intracellular
GO:0005615
extracellular space
GO:0031012
extracellular matrix
GO:0099512
supramolecular fiber
GO:0044424
intracellular part
GO:0043226
organelle
GO:0005578
proteinaceous extracellular matrix
GO:0005737
cytoplasm
GO:0044422
organelle part
GO:0043229
intracellular organelle
GO:0043228
non-membrane-bounded organelle
GO:0044444
cytoplasmic part
GO:0044446
intracellular organelle part
GO:0043232
intracellular non-membrane-bounded organelle
GO:0043292
contractile fiber
GO:0005694
chromosome
GO:0030016
myofibril
GO:0044449
contractile fiber part
GO:0044427
chromosomal part
GO:0030017
sarcomere
GO:0098687
chromosomal region
GO:0000775
chromosome, centromeric region

Figure S2

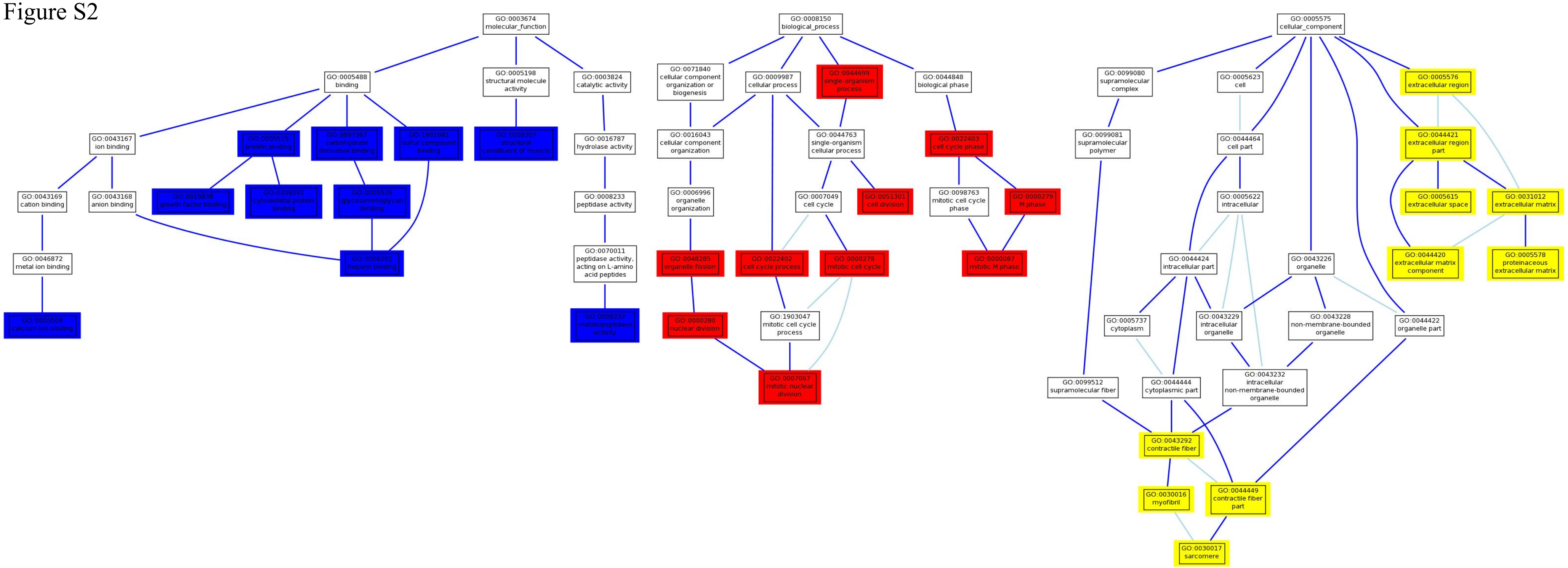

GO:0003674 molecular_function
GO:0005488 binding
GO:0005198 structural molecule activity
GO:0003824 catalytic activity
GO:0043167 ion binding
GO:0005515 protein binding
GO:0097367 carbohydrate derivative binding
GO:1901681 sulfur compound binding
GO:0008307 structural constituent of muscle
GO:0016787 hydrolase activity
GO:0043169 cation binding
GO:0043168 anion binding
GO:0019838 growth factor binding
GO:0008092 cytoskeletal protein binding
GO:0005539 glycosaminoglycan binding
GO:0008233 peptidase activity
GO:0046872 metal ion binding
GO:0008201 heparin binding
GO:0070011 peptidase activity, acting on L-amino acid peptides
GO:0005509 calcium ion binding
GO:0008237 metallopeptidase activity
GO:0008150 biological_process
GO:0071840 cellular component organization or biogenesis
GO:0009987 cellular process
GO:0044699 single-organism process
GO:0044848 biological phase
GO:0016043 cellular component organization
GO:0044763 single-organism cellular process
GO:0022403 cell cycle phase
GO:0006996 organelle organization
GO:0007049 cell cycle
GO:0051301 cell division
GO:0098763 mitotic cell cycle phase
GO:0000279 M phase
GO:0048285 organelle fission
GO:0022402 cell cycle process
GO:0000278 mitotic cell cycle
GO:0000087 mitotic M phase
GO:0000280 nuclear division
GO:1903047 mitotic cell cycle process
GO:0007067 mitotic nuclear division
GO:0005575 cellular_component
GO:0099080 supramolecular complex
GO:0005623 cell
GO:0005576 extracellular region
GO:0099081 supramolecular polymer
GO:0044464 cell part
GO:0044421 extracellular region part
GO:0005622 intracellular
GO:0005615 extracellular space
GO:0031012 extracellular matrix
GO:0044424 intracellular part
GO:0043226 organelle
GO:0044420 extracellular matrix component
GO:0005578 proteinaceous extracellular matrix
GO:0005737 cytoplasm
GO:0043229 intracellular organelle
GO:0043228 non-membrane-bounded organelle
GO:0044422 organelle part
GO:0099512 supramolecular fiber
GO:0044444 cytoplasmic part
GO:0043232 intracellular non-membrane-bounded organelle
GO:0043292 contractile fiber
GO:0030016 myofibril
GO:0044449 contractile fiber part
GO:0030017 sarcomere
